# Real-Time 6D Pose Estimation and Multi-Target Tracking for Low-Cost Multi-Robot System

**DOI:** 10.3390/s25237130

**Published:** 2025-11-21

**Authors:** Bo Shan, Donghui Zhao, Ruijin Zhao, Yokoi Hiroshi

**Affiliations:** 1 School of Electrical Engineering, Shenyang University of Technology, Shenyang 110178, China; 2 Jianghuai Advanced Technology Center, Hefei 230088, China; 3 Department of Mechanical Engineering and Intelligent Systems, University of Electro-Communications, Tokyo 182-8585, Japan

**Keywords:** multi-robot system, RGB sensing, 6D pose estimation, multi-target tracking, real-time perception, low-cost perception

## Abstract

In the research field of multi-robot cooperation, reliable and low-cost motion capture is crucial for system development and validation. To address the high costs of traditional motion capture systems, this study proposes a real-time 6D pose estimation and tracking method for multi-robot systems based on YolPnP-FT. Using only an Intel RealSense D435i depth camera, the system achieves simultaneous robot classification, 6D pose estimation, and multi-target tracking in real-world environments. The YolPnP-FT pipeline introduces a keypoint confidence filtering strategy (PnP-FT) at the output of the YOLOv8 detection head and employs Gaussian-penalized Soft-NMS to enhance robustness under partial occlusion. Based on these detection results, a linearly weighted combination of Mahalanobis distance and cosine distance enables stable ID assignment in visually similar multi-robot scenarios. Experimental results show that, at a camera height below 2.5 m, the system achieves an average position error of less than 0.009 m and an average angular error of less than 4.2°, with a stable tracking frame rate of 19.8 FPS at 1920 × 1080 resolution. Furthermore, the perception outputs are validated in a CoppeliaSim-based simulation environment, confirming their utility for downstream coordination tasks. These results demonstrate that the proposed method provides a low-cost, real-time, and deployable perception solution for multi-robot systems.

## 1. Introduction

With the ongoing advancement of automation and intelligence, multi-robot systems are being increasingly deployed in military, industrial, and agricultural applications. These systems critically rely on real-time and accurate state perception to provide essential 6D pose (position and orientation) and identity information for each robot. However, current mainstream motion capture (mocap) systems face significant limitations. Commercial optical mocap systems, such as Vicon [1], offer sub-millimeter accuracy but suffer from high cost, strong dependence on controlled environments (e.g., fixed cameras, no occlusion, stable lighting), and the requirement for specialized markers—severely restricting their scalability in open, dynamic scenarios. Meanwhile, markerless deep learning-based pose estimation methods, while more flexible, often exhibit sensitivity to partial occlusion and frequent identity switches (ID switches) in multi-target settings. Consequently, there is a pressing need for an integrated perception solution that balances accuracy, robustness, scalability, and cost-effectiveness.

In this context, robot localization methods can be broadly categorized into two paradigms: absolute and relative localization, each with distinct trade-offs. Relative localization relies on internal sensors [2] to estimate displacement and heading based on an initial pose. For instance, odometry using encoders and inertial sensors [3,4] is simple and low-cost, suitable for local navigation and short-term tasks; however, it is prone to cumulative errors and depends heavily on the initial pose. In contrast, absolute localization determines robot pose by recognizing environmental landmarks, making it suitable when the initial pose is unknown. LiDAR-based localization [5] provides accurate 3D positioning and is robust to lighting and electromagnetic interference [6], yet it incurs high computational overhead, lacks semantic information, requires global reference points for high accuracy, and entails substantial hardware costs. In practice, different localization strategies are selected based on application requirements. While multi-sensor fusion can combine the strengths of relative and absolute approaches to enhance information richness and estimation accuracy [7,8], it introduces new challenges, including strong dependency on model design, high computational demands for data alignment, increased system cost, and spatial deployment constraints.

Table 1 summarizes the key characteristics of representative visual localization methods. Current approaches either suffer from high cost and stringent environmental constraints (e.g., Vicon) or lack multi-target 6D tracking capability (e.g., GDR-Net, FairMOT). Particularly in visually homogeneous and resource-constrained multi-robot scenarios, there remains a critical gap for an integrated perception solution that simultaneously ensures 6D accuracy, ID stability, and deployment affordability—this constitutes the core motivation of our work.

To address these challenges, this study proposes YolPnP-FT, a low-cost, and deployable integrated perception system tailored for top-down multi-robot scenarios. We do not introduce a novel 6D pose estimation algorithm or a new tracking architecture. Instead, we integrate and optimize existing components—YOLOv8, a keypoint confidence filtering strategy (PnP-FT), and DeepSORT—to construct an end-to-end pipeline for multi-target 6D pose estimation, tracking, and task-level validation. The main contributions are summarized as follows:

- We present a low-cost semi-physical verification platform for multi-robot systems, integrating an affordable RGB-D camera (Intel D435i), a standard GPU workstation, and multiple Mecanum-wheeled robots. This setup supports closed-loop validation from real-world perception to simulation-based control, lowering the barrier for algorithm development and system testing.
- We propose the YolPnP-FT perception pipeline, which introduces a keypoint confidence filtering strategy (PnP-FT) after the YOLOv8 detection head. This strategy effectively enhances the robustness of PnP-based pose estimation under partial occlusion while reducing unnecessary computation.
- We adapt the DeepSORT tracker by employing a linearly weighted combination of Mahalanobis distance and cosine distance with cascaded matching. This strategy enables the system to achieve stable ID assignment in visually similar multi-robot scenarios, effectively mitigating tracking instability and identity confusion caused by occlusion or appearance homogeneity.
- We establish a perception-driven validation paradigm: real-time perception outputs (6D pose + ID) are directly injected into a CoppeliaSim simulation environment to verify their direct utility in downstream tasks such as formation control and path planning, offering a reproducible deployment-validation pathway for low-cost systems.

In summary, this paper presents a low-cost, deployable 6D perception system for specific multi-robot applications. By integrating and optimizing existing techniques, we evaluate pose accuracy and tracking stability in real-world environments and validate task-level performance through simulation-based closed-loop testing. Experimental results demonstrate that the proposed solution achieves an effective balance between affordability and practicality.

The remainder of this paper is organized as follows: Section 2 reviews related work; Section 3 details the proposed methodology; Section 4 presents experimental results and analysis; and Section 5 concludes the study.

## 2. Related Works

Achieving robust and efficient perception in complex environments is essential for enhancing the adaptability and collaborative performance of multi-robot systems. In this context, 6D pose estimation and multi-object tracking (MOT) serve as two foundational pillars. While significant progress has been made in each subfield independently, there remains a critical gap in integrated solutions tailored for visually homogeneous, resource-constrained multi-robot scenarios.

### 2.1. Six-Dimensional Pose Estimation

Six-dimensional pose estimation is a key enabler for precise navigation and interaction in multi-robot systems. Recent advances in deep learning have substantially advanced this field. PoseNet [13] was among the first to employ convolutional neural networks (CNNs) for end-to-end 6D pose regression directly from RGB images, demonstrating the feasibility of learning-based approaches. PoseCNN [14] introduced direct pose regression but suffered from limited generalization and accuracy. Deep-6Dpose [15], built upon Mask R-CNN, added a dedicated pose prediction branch to streamline the end-to-end pipeline. YOLO-6D [16] reformulated pose estimation as a keypoint regression task within the real-time YOLOv2 framework, achieving high inference speed but exhibiting sensitivity to symmetric objects and partial occlusion. To better handle occlusion, ZebraPose [17] proposed a dense surface representation using discrete descriptors, showing improved robustness under controlled benchmarks. EfficientPose [18] reduced computational overhead by predicting translation and rotation through auxiliary sub-networks, though its performance degraded under severe occlusion. GDR-Net [10] introduced a geometry-guided approach that leveraged dense correspondences as an intermediate representation, achieving state-of-the-art results in the BOP2022 challenge; however, it required extensive post-processing and was not designed for real-time deployment. DPOD [19] combined detection with dense matching for single-RGB pose estimation and demonstrated robustness to natural occlusions. RNNPose [20] employed recurrent networks for iterative refinement, while PointNet++ [21] learned geometric features directly from point clouds but incurred high computational cost. PVN3D [11] and DenseFusion [22] adopted two-stage pipelines—using least-squares fitting or voting mechanisms—and achieved strong performance on benchmarks like YCB-Video.

However, these methods share a common limitation: they primarily focus on single-object pose estimation [23]. None have explicitly addressed the challenges of multi-target association or maintaining temporal identity consistency—capabilities that are crucial for algorithm-driven multi-robot cooperation in intelligent task allocation and formation planning.

### 2.2. Multi-Object Tracking (MOT)

In multi-robot systems, MOT ensures consistent cross-frame identity assignment and real-time localization, which are critical for collaborative tasks. JDE [24] proposed an end-to-end framework that jointly performs detection and feature embedding, reducing system complexity. FairMOT [12] adopted a two-stage architecture with a “fair” matching strategy to handle occlusion and appearance changes. CenterTrack [25] relied on center-point association for fast tracking, while ChainedTracker [26] used graph-based modeling to resolve target associations in highly interactive scenarios. SORT [27] combined Kalman filtering with IoU-based matching, offering simplicity and efficiency but suffering from frequent ID switches under occlusion. DeepSORT [13] extended SORT by incorporating deep appearance descriptors and a cascaded matching strategy, significantly improving ID stability in pedestrian tracking benchmarks [28].

Nevertheless, existing MOT frameworks exhibit two major limitations in the context of multi-robot perception: They typically output only 2D bounding boxes or centroids, without providing 6D pose information; they heavily rely on appearance diversity for ReID feature learning. In multi-robot systems, robots often share visually homogeneous designs (e.g., similar chassis, color schemes), causing ReID features to lack discriminative power and leading to severe tracking failures in practice [29].

### 2.3. Research Gap

In summary, although both 6D pose estimation and MOT have advanced considerably, existing works largely optimize single-object pose accuracy or ID continuity in isolation. This fragmented approach fails to meet the integrated demands of resource-constrained, visually homogeneous multi-robot scenarios.

To address this gap, this study proposes a pragmatic integration of YOLOv8, a keypoint-based PnP solver with confidence filtering (PnP-FT), and an adapted DeepSORT tracker. Our system is designed for single top-down RGB-D camera setups, prioritizing deployability and reliability in real-world conditions over benchmark-leading performance. The primary goal is not to surpass SOTA methods on generic datasets, but to deliver a coherent, low-cost perception pipeline that enables robust 6D tracking for visually similar robots—a capability currently missing in the literature.

## 3. Method

### 3.1. System Overview

The proposed low-cost perception system for multi-robot formation validation comprises two complementary components:

(1) A physical hardware platform for real-time 6D pose estimation and tracking, consisting of an Intel RealSense D435i RGB-D camera (Santa Clara, CA, USA), a commercial GPU workstation, and multiple heterogeneous Mecanum-wheeled robots;
(2) A simulation validation environment based on CoppeliaSim, which receives the 6D pose and identity (ID) outputs from the physical perception system to drive virtual robot models.

**Figure 1:**
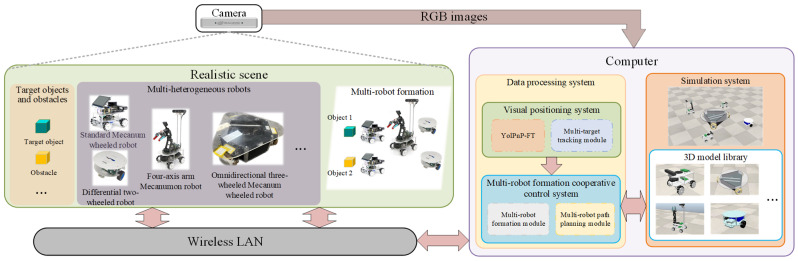
System Architecture for Perception-Driven Multi-Robot Validation: Real-world 6D pose and ID estimates from the YolPnP-FT pipeline (D435i camera) are injected into CoppeliaSim to drive virtual robots for downstream task validation.

This design establishes a perception-driven validation mechanism: the perception system operates independently in the real world, and its outputs are fed into the simulation environment to enable safe and reproducible verification of downstream coordination algorithms (e.g., formation control, path planning). The overall system architecture is illustrated in Figure 1, which clearly depicts the inter-component relationships and data flow.

### 3.2. A 6D Pose Estimation Method Based on the YolPnP-FT

This section introduces the proposed YolPnP-FT perception pipeline, with heterogeneous Mecanum-wheeled robots selected as targets due to their low cost, relatively complex kinematics, and visual homogeneity in multi-robot scenarios. The pipeline implementation consists of three sequential stages: (1) dataset construction and network training; (2) real-time 6D pose estimation using the trained YolPnP-FT network; (3) multi-object tracking to maintain cross-frame ID consistency.

#### 3.2.1. Dataset Construction

This subsection details the dataset construction process for Mecanum-wheeled robots. The dataset focuses on typical indoor collaborative scenarios rather than extreme lighting or background clutter. Specifically, 700 images covering all semantic keypoints were collected under typical office lighting conditions (300–800 lux) using an Intel D435i camera mounted at heights of 0.5–2.5 m to simulate practical deployment scenarios.

**Figure 2:**
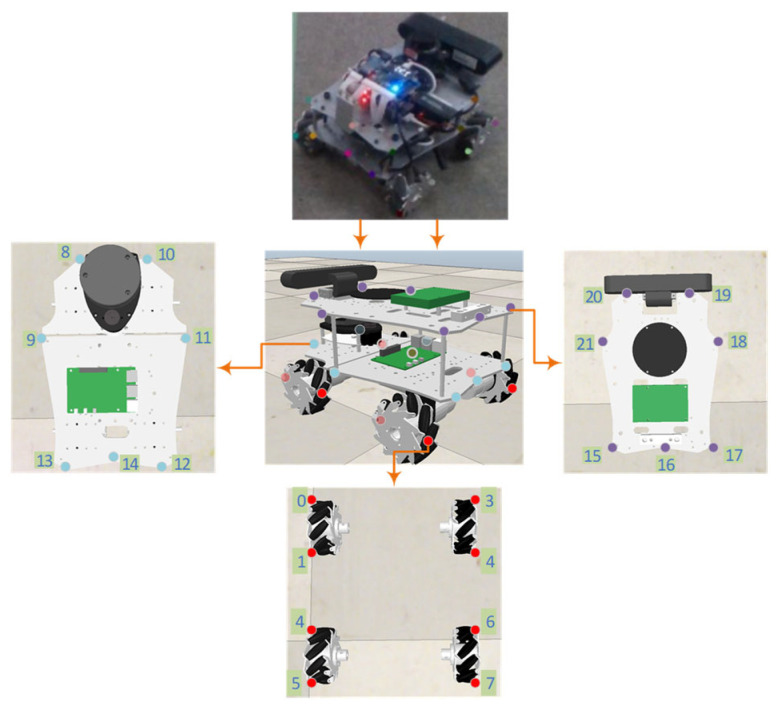
Example of 2D-3D Keypoint Correspondence: Illustration of the 22 semantic keypoints (e.g., wheel hub centers, arm joints) annotated on a Mecanum-wheeled robot under a top-down view, derived from its 3D CAD model.

For each robot type, a 3D CAD model was reconstructed in a virtual environment, and 22 semantic keypoints were defined to capture structural features (e.g., wheel hub centers, robotic arm joints). These 2D keypoints were manually annotated on extracted keyframes using Labelme, ensuring sufficient visibility under typical top-down viewpoints (see Figure 2 for an annotation example). Approximately 30% of the frames exhibit partial occlusion due to inter-robot occlusion, realistically reflecting multi-robot operational conditions.

#### 3.2.2. Six-Dimensional Pose Estimation Based on the YolPnP-FT Perception Pipeline

**Figure 3:**
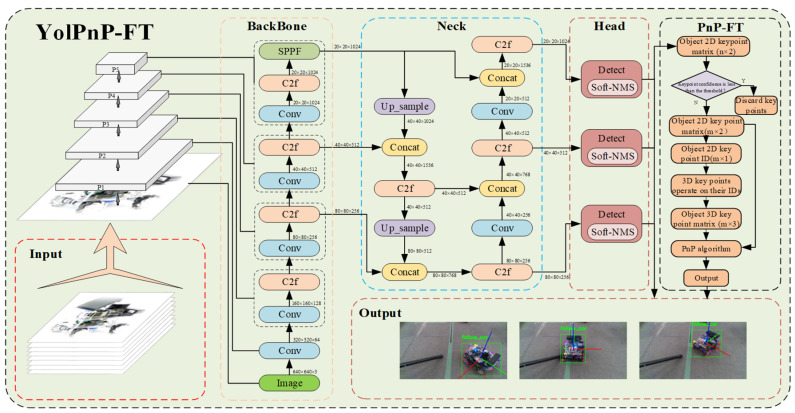
Network Architecture for 6D Pose Analysis Based on YolPnP-FT.

This subsection details the proposed method for multi-robot 6D pose estimation. We formulate the YolPnP-FT perception pipeline as an integrated system for multi-object detection and 6D pose estimation, comprising three core components: a YOLOv8 detector, Gaussian-penalized Soft-NMS, and a non-learnable keypoint confidence filtering strategy (Perspective-n-Point with Filtered Threshold, PnP-FT). The overall architecture is illustrated in Figure 3.

**Figure 4:**
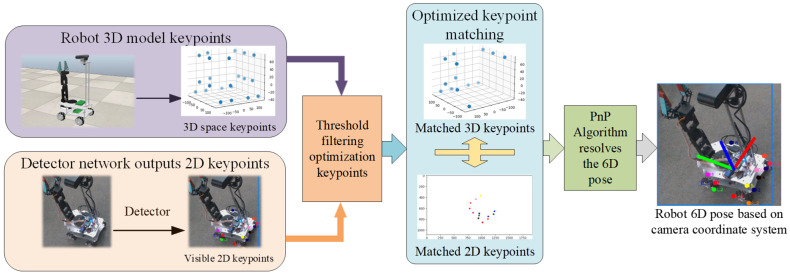
Schematic Diagram of the Keypoint Matching Algorithm for PnP-FT 6D Pose Estimation.

As shown in Figure 3, the input consists of video frame sequences. The Backbone employs an enhanced C2F module for feature extraction, the Neck performs multi-scale feature fusion, and the Head adopts a decoupled design to separately handle localization and classification tasks. In contrast to the standard YOLOv8 network—which is specialized for object detection and outputs class labels, keypoints, and segmentation masks—we introduce a lightweight, non-learnable PnP-FT strategy module immediately after the detection head. This module filters out low-confidence keypoints prior to PnP-based pose estimation, thereby enhancing robustness under partial occlusion. The keypoint matching workflow of the PnP-FT strategy is illustrated in Figure 4.

To address the frequent keypoints occlusion caused by complex robot configurations and top-down camera viewpoints—particularly in dense multi-robot formations where bounding box overlap (IoU > 0.5) is common—we embed Gaussian-penalized Soft-NMS [30] into the detection head, replacing conventional hard NMS or linear Soft-NMS. In such scenarios, hard NMS tends to over-suppress overlapping detections, while linear Soft-NMS (Equation (1)) causes abrupt score decay that destabilizes downstream keypoint matching. In contrast, Gaussian-penalized Soft-NMS (Equation (2)) provides smooth confidence attenuation, preserving recall while maintaining score discriminability—critical for the stability of PnP-FT inputs. This design aligns with practices in recall-sensitive pose estimation systems (e.g., EfficientPose [14]).

Specifically, candidate detection boxes are first sorted in descending order of confidence. The box with the highest confidence serves as the reference. The IoU between this reference box and all other candidates is then computed. When the IoU exceeds a preset threshold, the confidence of the overlapping box is decayed using the Gaussian Soft-NMS formulation. If the decayed confidence falls below a secondary threshold, the candidate is discarded. This process iterates until all boxes are processed.

(1) $$s_{j}^{’}=\left\{\begin{array}{ll} s_{j} & \mathrm{if}\;\mathrm{IoU}(b_{i},b_{j})<{\mathrm{IoU}}_{\mathrm{threshold}}\\ s_{j}\times (1-\mathrm{IoU}(b_{i},b_{j})) & \mathrm{otherwise} \end{array}\right.$$

(2) $${s^{\prime }}_{j}=s_{j}\exp\left(-\frac{{\mathrm{IOU}}^{2}(b_{j},b_{i})}{\sigma }\right)$$

$s_{j}^{’}$ represents the adjusted confidence score, $s_{j}$ is the original confidence score, $b_{j}$ denotes overlapping candidate boxes, $b_{i}$ is the current box, and σ is a hyperparameter controlling the rate of score decay. Linear penalty is suitable for simple scenarios with high real-time performance and limited resources, whereas Gaussian penalty is better for complex scenes requiring high-precision detection. Given the complexity of multi-robot systems and precision needs, we choose Gaussian penalty to maintain detection continuity, avoid sudden score drops, improve recall rates, and enhance system robustness.

Due to occlusion induced by camera angles and robot formations, traditional PnP algorithms often suffer from degraded performance. To mitigate this, we propose a geometry-aware keypoint filtering strategy (formulated in Equations (5) and (6)) that evaluates whether the relative positions among neighboring keypoints conform to the robot’s known 3D structural prior. Invalid or structurally implausible keypoints are discarded before PnP pose estimation, thereby improving both accuracy and stability.

**Figure 5:**
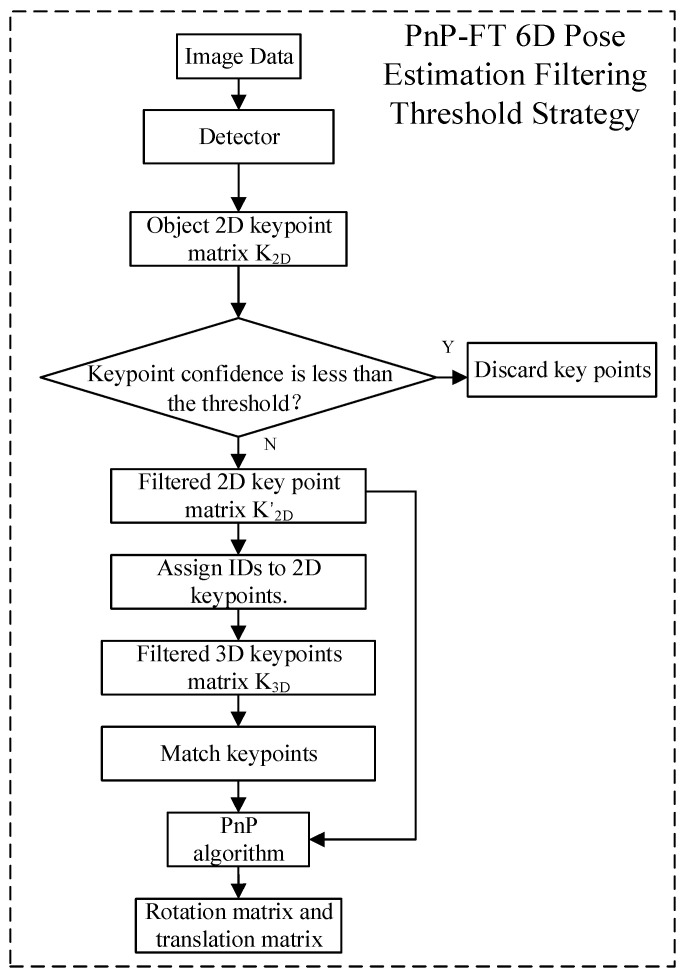
Keypoint Confidence Filtering Strategy (PnP-FT) in the YolPnP-FT Pipeline.

Figure 5 illustrates the computational workflow of the PnP-FT 6D pose estimation algorithm, with its corresponding pseudocode provided in Algorithm 1. Beginning with the image data captured by camera, each frame of image data, denoted as I, is processed by a trained keypoint detector to extract 2D keypoints $p_{i}\left(X_{c},Y_{c},Z_{c}\right)$ from the current video frame. These keypoints form a keypoint matrix, as shown in Equation (3).

(3) $$K_{2D}=D(I)$$

Subsequently, to ensure the reliability of the 2D keypoints, the confidence of each keypoint is evaluated. A confidence threshold, denoted as τ, is applied, and only keypoints with a confidence higher than the threshold are retained, forming a filtered keypoint matrix $K_{2D}^{’}$ (Equation (4)) as follows.

(4) $${K^{’}}_{2D}=\left\{k_{2D}^{\left(i\right)}|C\left(k_{2D}^{\left(i\right)}\right)\geq \tau \right\},i=1,2,\ldots ,n$$

The confidence function $C\left(k_{2D}^{\left(i\right)}\right)$ is defined to assess and filter the confidence of the 2D keypoints. This function takes into account the response strength of keypoint detection as well as the geometric consistency with adjacent keypoints. The specific definition is as shown in Equation (5):

(5) $$C(k_{2D}^{\left(i\right)})=\alpha \cdot S(k_{2D}^{\left(i\right)})+\beta \cdot G(k_{2D}^{\left(i\right)})$$

Here, α and β are weighting coefficients. The parameter α controls the tolerance to inter-keypoint deviations: higher values impose stricter geometric consistency constraints by penalizing larger displacements, whereas lower values allow more lenient matching. α should be adaptively tuned according to the actual keypoint distribution in the scene; in cases with significant appearance variations (e.g., due to lighting changes or texture ambiguity), increasing α can help prioritize reliable detection confidence over geometric fit. The parameter β governs the weight of geometric consistency in the overall association score. A higher β emphasizes the spatial configuration of keypoints, thereby enhancing robustness to challenging conditions such as occlusion or viewpoint changes—common in multi-robot interaction scenarios.
**Algorithm 1:** PnP-FT 6D Pose Estimation Filtering Threshold Strategy**Input**: *Image data I, 3D keypoint model K_3D_ confidence threshold θ***Output**: *Rotation matrix R, translation vector t*1K_2D_ ← Detector(*I*);
*// Detect 2D keypoints from image*2**if** confidence (*k*) < **for any** *k* ∈ $K_{2D}$ **then**3  Discard low-confidence keypoints;4    $K_{2D}^{’}$ ← {k∈$K_{2D}$| confidence(k)≥*θ*};5Assign IDs to keypoints in $K_{2D}^{’}$
6$K_{3D}^{filtered}\;$← GetMatching 3D Keypoints ($K_{2D}^{’}$, $K_{3D})$;
// Select corresponding 3D keypoints7Match keypoints between $K_{2D}^{’}$ and $K_{3D}^{filtered}$;8[R,t] ← PnP($K_{2D}^{’}$, $K_{3D}^{filtered}$);
// Solve for pose using PnP algorithm9**return** R,t;

$S(k_{2D}^{\left(i\right)})$ represents the keypoint detection response strength—i.e., the raw confidence score. However, relying solely on the detection response strength $S\left(k_{2D}^{\left(i\right)}\right)$ is often insufficient to reliably assess keypoint quality. For example, under partial occlusion, the network may output keypoints with high confidence but significantly inaccurate locations. To address this limitation, we introduce a geometric consistency constraint $G\left(k_{2D}^{\left(i\right)}\right)$, which evaluates whether the relative positions between a keypoint and its neighboring keypoints conform to the robot’s 3D structural prior, thereby filtering out structurally implausible keypoints. Considering that keypoint errors in real-world scenarios typically follow a distribution where “small deviations are common, while large structural distortions are rare,” we model $G\left(k_{2D}^{\left(i\right)}\right)$ using an exponential decay function. This formulation remains tolerant to minor positional deviations while being highly sensitive to significant geometric distortions, effectively suppressing the influence of outlier keypoints on subsequent 6D pose estimation.

The geometric consistency function is designed as shown in Equation (6):

(6) $$G(k_{2D}^{\left(i\right)})=\exp\left(-\frac{1}{\left|N\right|}\sum_{k_{2D}^{\left(j\right)}\in N}\frac{\left\|k_{2D}^{\left(i\right)}-k_{2D}^{\left(j\right)}\right\|-d_{ij}}{\sigma }\right)$$

This approach ensures that only keypoints with high confidence are utilized in subsequent pose estimation and tracking processes, enhancing the robustness and accuracy of the system. Where N represents the neighborhood set of keypoint $k_{2D}^{\left(i\right)}$, $d_{ij}$ is the desired distance between keypoints, and σ is a scale parameter that controls the sensitivity of geometric consistency. $\left\|{k_{2D}^{\left(i\right)}-k}_{2D}^{\left(j\right)}\right\|$ is the Euclidean distance between keypoint $k_{2D}^{\left(i\right)}$ and its adjacent keypoint $k_{2D}^{\left(j\right)}$. By adjusting the weight coefficients α and β, as well as the parameters $d_{ij}$ and σ in geometric consistency, the method can be flexibly adapted to different application scenarios.

This approach filters out low-confidence keypoints, thereby improving the accuracy and robustness of pose estimation. Following confidence filtering, the retained 2D keypoints are assigned IDs, and the corresponding 3D keypoints $P_{i}\left(X_{w},Y_{w},Z_{w}\right)$ are matched to form a matched 3D keypoint matrix $K_{3D}$.

**Figure 6:**
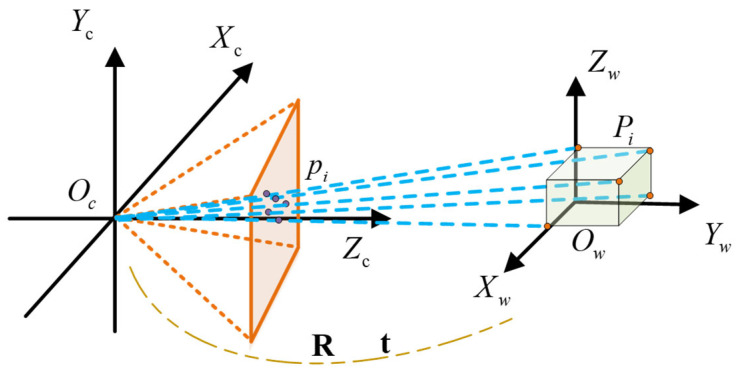
Schematic Diagram of the Camera Coordinate System and World Coordinate System Model.

In summary, the threshold-based filtering and matching yield 2D keypoint matrix $K_{2D}^{’}$ composed of 2D keypoints $p_{i}\left(X_{c},Y_{c},Z_{c}\right)$ and 3D keypoint matrix $K_{3D}$ composed of 3D keypoints $P_{i}\left(X_{w},Y_{w},Z_{w}\right)$. Subsequently, the PnP algorithm employing the DLT (Direct Linear Transform) method is utilized. As illustrated in Figure 6, the re-projection equation is derived based on the camera projection model and the pose relationship between the camera coordinate system and the world coordinate system, which can be expressed as shown in Equation (7):

(7) $$p_{i}=K\left[R|t\right]P_{i}\;\;i=1,\ldots ,n$$

where K is the intrinsic matrix of the camera obtained through Zhang’s calibration method. The coordinates $P_{i}\left(X_{w},Y_{w},Z_{w}\right)$ in the world coordinate system can be represented as homogeneous coordinates ${\left[\begin{array}{cc} X_{w} & Y_{w} \end{array}\begin{array}{cc} Z_{w} & 1 \end{array}\right]}^{T}$ and the 2D keypoint $p_{i}\left(X_{c},Y_{c},Z_{c}\right)$ in the image coordinate system can be represented as homogeneous coordinates ${\left[\begin{array}{ccc} u & v & 1 \end{array}\right]}^{T}$. The equation can be rearranged as shown in Equation (8):

(8) $$\lambda \left[\begin{array}{c} u_{c}\\ v_{c}\\ 1 \end{array}\right]=M\left[\begin{array}{c} X_{w}\\ Y_{w}\\ Z_{w}\\ 1 \end{array}\right]$$

where λ is the scaling factor, and M is the projection matrix, obtained by the product of the intrinsic matrix k and the extrinsic matrix [R|t]. The extrinsic parameters can be separated through inversion, as shown in Equation (9):

(9) $$\left[R|t\right]=K^{-1}\cdot M$$

To ensure that R is a valid rotation matrix, perform a singular value decomposition (SVD) on the 3 × 3 matrix R, as shown in Equation (10).

(10) $$R=U\cdot \sum \cdot V^{T}$$

Further, to extract the translation vector t, as shown in Equation (11), multiply the inverse of the intrinsic matrix $K^{-1}$ by the fourth column of M:

(11) $$t=K^{-1}\cdot M\left[:,4\right]$$

This method effectively addresses the limitations of traditional pose estimation techniques in handling partially occluded or invisible key points. By eliminating redundant or unreliable keypoints, it reduces computational load and enhances robustness in complex real-world environments.

#### 3.2.3. Adapted DeepSORT for Multi-Object Tracking in Visually Homogeneous Multi-Robot Scenarios

The classic multi-object tracking algorithm SORT combines Kalman filtering with the Hungarian algorithm, using Intersection over Union (IoU) as the sole association metric. While computationally efficient, this approach is prone to frequent identity switches (ID switches) when targets undergo occlusion or temporarily leave and re-enter the field of view—common occurrences in complex multi-robot scenarios.

To address this limitation, we adapt the DeepSORT tracking framework, using the YolPnP-FT pipeline as the detector. Specifically, we employ a linearly weighted combination of Mahalanobis distance and cosine distance within a cascaded matching strategy to enhance ID stability in visually homogeneous multi-robot environments and provide reliable state estimates for downstream coordination tasks.

Although single-stage trackers such as FairMOT [23] offer end-to-end efficiency, they heavily rely on appearance diversity for ReID feature learning. In multi-robot systems, where robots often exhibit high visual similarity (e.g., identical chassis, color schemes, and structural symmetry), ReID features lack discriminative power, leading to degraded tracking performance. In contrast, DeepSORT’s cascaded matching mechanism explicitly fuses motion and appearance cues, making it better suited for scenarios with low appearance variance—a characteristic inherent to visually similar robot fleets.

(1) Motion Model Information Feature Matching: Mahalanobis Distance

Using the multi-object bounding boxes from the YolPnP-FT detected, Mahalanobis distance enhances tracking stability by considering the covariance between data points, reducing scale variation effects. Therefore, the matching degree Equation (12) between Trajectory and Detection is as follows:

(12) $$d^{\left(1\right)}(i,j)={\left(d_{j}-y_{i}\right)}^{T}S_{i}^{-1}(d_{j}-y_{i})$$

(13) $$b_{i,j}^{\left(1\right)}=1[d^{\left(1\right)}(i,j)\leq t^{\left(1\right)}]$$

The Mahalanobis distance $d^{\left(1\right)}(i,j)$ represents the motion matching degree between the state $d_{j}$ of the j-th detection and the predicted observation $y_{i}$ of the i-th trajectory at the current moment. $S_{i}^{-1}$ represents the inverse covariance matrix predicted by the Kalman filter. The association between the tracking box and the detection box in terms of motion information is determined by Equation (13).

Considering the continuous motion of dynamic targets, the distance between consecutive frames remains small, allowing similar coordinate boxes to be identified as the same target. A threshold t = 0.95 is used to filter detections. If the distance between the detection box and the tracking box is less than t, they are considered associated, setting b = 1; otherwise, b = 0.

(2) Appearance Model Information Feature Matching: Cosine Distance

While the Mahalanobis distance is effective for well-defined target motions, real-world conditions such as camera movement and target uncertainty can degrade Kalman filter performance. When targets are close or occluded, bounding box distance and Mahalanobis distance become limitation. To alleviate this problem, the YolPnP-FT outputs is used to extract feature vectors, and cosine similarity is employed to quantify the feature differences between targets in adjacent frames. The calculation equation is as follows:

(14) $$d^{\left(2\right)}(i,j)=\min\{1-r_{j}^{T}r_{k}^{\left(i\right)}|r_{k}^{\left(i\right)}\in R_{i}\}$$

(15) $$R_{i}={\left\{r_{k}^{\left(i\right)}\right\}}_{k=1}^{L_{k}}$$

$d^{\left(2\right)}(i,j)$ (Equation (14)) represents the minimum cosine distance between the feature vector of the tracked target and the feature vector of the detected target in the current frame within set $R_{i}$. $r_{j}$ denotes the appearance feature vector of the j-th detected result, and $R_{i}$. (Equation (15)) is the set of the most recent $L_{k}$ feature vectors of the i-th tracked target. The cosine similarity $r_{j}^{T}r_{k}^{\left(i\right)}$, measures the appearance information, improving target ID prediction accuracy.

(16) $$b_{i,j}^{\left(2\right)}(i,j)=1[d^{\left(2\right)}(i,j)\leq t^{\left(2\right)}]$$

The threshold in the cosine metric is set according to Equation (16). When the metric value falls below t^(2)^, we consider the detection box and the tracking box content to be successfully matched. Using cosine distance for correlation analysis can effectively address uncertainties in motion and camera vibrations, thus reducing the interference of external environments on system stability.

In summary, appearance information feature solves some limitations of Mahalanobis distance in target tracking, which makes the appearance information feature better at long-term tracking.

(3) Comprehensive Matching Degree

Combining the motion model and appearance model through linear weighting complements their strengths and weaknesses to form the final matching score. The combined score equation is as follows:

(17) $$c(i,j)=\lambda d^{\left(1\right)}(i,j)+(1-\lambda )d^{\left(2\right)}(i,j)$$

The combined matching score c(i,j) uses a linear weighting coefficient λ to bring both distance and feature metrics close to their minimum values. When motion and appearance information correlate, a link is established between tracking and detection boxes. The final association is defined by Equation (18).

(18) $$b_{i,j}=\prod_{m=1}^{2}b_{i,j}^{\left(m\right)}$$

When the indicator b equals 1, the initial match is considered successful. The combined measurement leverages the strengths of both methods, considering both the distance similarity between bounding boxes and the content similarity within them.

(4) Cascade Matching Strategy

**Figure 7:**
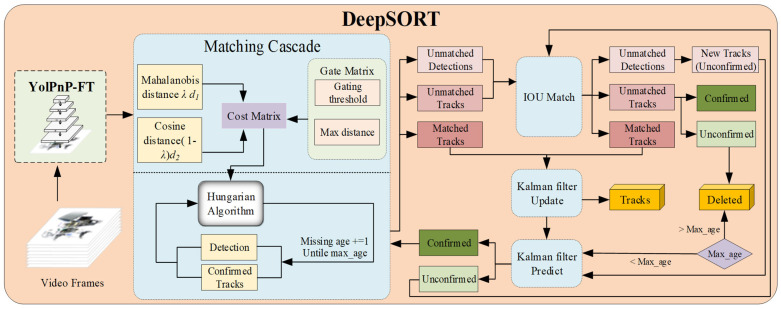
Architecture Diagram of the DeepSort Algorithm.

When targets are occluded for long periods, the performance of the Kalman filter is limited, leading to discontinuities in the probability of consecutive predictions. To improve the accuracy of the matches, we introduce a cascaded matching strategy. The cascaded matching strategy consists of two parts: upper and lower, as illustrated in Figure 7. In the upper part, using the motion model to calculate the distance between the bounding boxes of tracks and observations, construct the cost matrix. By linearly weighting $d^{\left(1\right)}\left(i,j\right)$ and $d^{\left(2\right)}(i,j)$, obtain the combined matching score c(i,j). A threshold matrix is used to limit the higher values in the cost matrix. In the lower part, starting with tracks that have not been lost, prioritize these tracks and match tracks lost for the longest last. Unconfirmed tracks and those consistently tracked are matched with detection results based on Intersection over Union (IOU) to reduce matching errors caused by sudden appearance changes or partial occlusions. Throughout the process, matching priority is dynamically adjusted, with more frequently appearing targets receiving higher priority. In summary, unlike SORT, DeepSORT combines motion and appearance information for association, using cascade matching + IOU matching to enhance tracking accuracy and robustness. The algorithm steps are shown in the algorithm flowchart in Algorithm 2.
**Algorithm 2:** DeepSORT Algorithm**Input**: *Frame *$F_{t}$*, detections *D *= (*$d_{1}$*,,...,* $d_{M}$*), track set T, max age *$A_{max}$*, IOU threshold* λ**Output**: *Updated track set *T1**for*** each track *$\tau_{i}$ ∈ T **do**2
  
$\tau_{i}\;$. predict() *// Kalman prediction*
*// Stage 1: Cascade Matching (appearance +motion)*3$T_{conf}\;$← {$\tau_{i}$ ∈ T ∣ $\tau_{i}$.is_confirmed()};4$M_{1}$, $U_{T}$, $U_{D}$ ← ∅, $T_{conf}$, D;5**for** n = 1 **to** $A_{max}$
**do**6
  $T_{n}$ ← {$\tau_{i}$∈ $U_{T}$∣$\tau_{i}$. age = n};7
  **if** $T_{n}$ ≠ ∅ **then**8
  Compute cost matrix C using appearance + Mahalanobis;9
  [$x_{ij}$] ← Hungarian(*C*);10  $M_{n}$ ← {(i,j)∣$x_{ij}$ = 1 and $c_{ij}$ < gate};11  $M_{1}$ ← $M_{1}$ ∪ $M_{n}$;12  $U_{T}$ ← $U_{T}$\{i∣(i,j) ∈$\;M_{n}$};13  $U_{D}$ ←$\;U_{D}$\{j∣(i,j) ∈ $M_{n}$};
*// Stage2: IOU Matching for remaining confirmed tracks*14$M_{2}$, $U_{T}$, $U_{D}$ ← IOUMatch($U_{T}$, $U_{D}$, λ);
*// Update matched tracks*15**for** each (i,j) ∈ M_1_ ∪ M_2_
**do**16       $\tau_{i}$. update($d_{j}$);17       $\tau_{i}$. age ← 0;
*// Handle unmatched confirmed tracks*18**for** each $\tau_{i}$ ∈ $U_{T}$where $\tau_{i}$.is_confirmed() **do**19       $\tau_{i}$ . age ← $\tau_{i}$. age + 1;20       **if**
$\tau_{i}$.age > $A_{max}$
**then**21       T ← T\{$\tau_{i}$};
*// Create new unconfirmed tracks*22**for** each $d_{j}$ ∈ $U_{D}$
**do**23       $\tau_{new}$← newTrack($d_{j}$);24       $\tau_{new}$ . confirmed ← false;25       $\tau_{new}$ . age ← 1;26       T ← T ∪ {$\tau_{new}$};
*// Manage unconfirmed tracks*27**for** each $\tau_{i}$ ∈ T where ¬$\tau_{i}$.is_confirmed() **do**28       **if** $\tau_{i}$.age > 1 **then**29       T ← T\{$\tau_{i}$} *// Delete if not confirmed in 2 frames*30**return** T;

### 3.3. Downstream Task Validation of Perception Outputs

To validate the practical utility of the YolPnP-FT perception pipeline outputs, we inject the real-time 6D pose and identity ID information into a CoppeliaSim-based simulation environment, where they serve as state inputs for downstream coordination tasks such as formation control and path planning. This setup provides an intuitive and user-friendly interface for closed-loop algorithm validation without requiring physical robot deployment.

It is emphasized that the control and planning algorithms employed in simulation—namely, the leader–follower formation controller and the RRT path planner—are standard methods and do not constitute contributions of this work. Their sole purpose is to act as consumers of the perception outputs, thereby verifying whether YolPnP-FT provides sufficiently accurate and stable state estimates to support real-world multi-robot coordination.

As illustrated in Figure 1, the RGB-D data stream from the Intel D435i camera is fed into the YolPnP-FT perception pipeline. The resulting 6D pose and ID estimates are transmitted to CoppeliaSim via a TCP/IP interface to drive virtual robot models. Consequently, the observed performance in simulation—such as formation stability and path feasibility—directly depends on the pose accuracy and ID consistency delivered by YolPnP-FT, thereby enabling task-level validation of the perception system.

## 4. Experiment and Analysis

### 4.1. Real-Time 6D Pose Estimation and Tracking Experiments for Multi-Robot Formations Based on the YolPnP-FT Perception Pipeline

This section presents a systematic evaluation of the proposed YolPnP-FT perception pipeline in real-world multi-robot scenarios. The experimental procedure consists of three stages: (1) construction of a robot-specific dataset and training of the detection network; (2) 6D pose estimation using the trained YolPnP-FT, with validation through comparison between ground-truth and estimated poses; (3) real-time multi-robot formation tracking experiments.

#### 4.1.1. Experimental Environment

All experiments are implemented using the PyTorch deep learning framework on a Windows 10 system. The hardware configuration includes an NVIDIA GeForce RTX™ 3060 GPU (12 GB VRAM), an Intel^®^ Core™ i7-12700 CPU (up to 4.90 GHz), and 32 GB RAM. Visual data are captured using an Intel^®^ RealSense™ D435i depth camera, with only the RGB stream serving as input to the YolPnP-FT pipeline.

#### 4.1.2. 6D Pose Estimation Experiments Based on the YolPnP-FT Perception Pipeline

**Figure 8:**
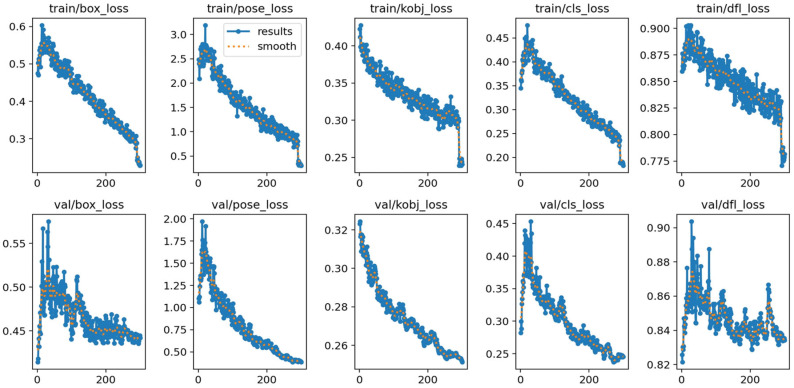
The Loss Function Curve of Network Training.

Following the dataset construction procedure described in Section 3.2.1, the network is trained with a learning rate of 0.001, batch size of 16, IoU threshold of 0.5, and 300 training epochs. The robot keypoint dataset is split into training and validation sets at a 5:1 ratio. The training loss curve is shown in Figure 8. The loss decreases rapidly in early epochs and stabilizes after approximately 200 iterations, though minor fluctuations are observed. These fluctuations are typical in keypoint detection tasks and primarily stem from partial keypoint occlusion in certain frames, which leads to discontinuous supervision signals [Ultralytics, YOLOv8 Docs] [31]. Nonetheless, the overall convergence behavior confirms the effectiveness of the optimization process.

**Figure 9:**
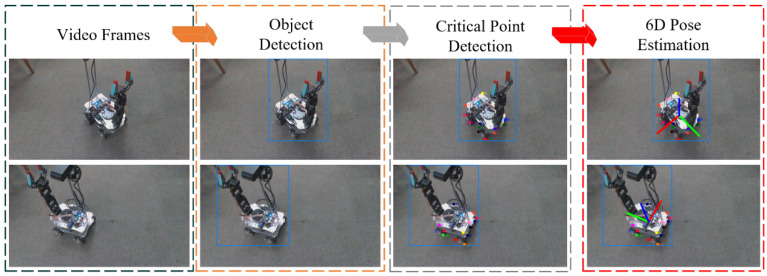
Illustrative Diagram of Staged Outputs from the YolPnP-FT Network.

The best-performing weights from training are deployed in the YolPnP-FT pipeline. To evaluate model performance, a video sequence of a four-axis Mecanum-wheeled robot is processed, and intermediate outputs—including object detection, keypoint localization, and 6D pose estimation—are extracted programmatically (Figure 9). Results demonstrate that the pipeline effectively achieves simultaneous object classification, keypoint detection, and full 6D pose estimation.

**Figure 10:**
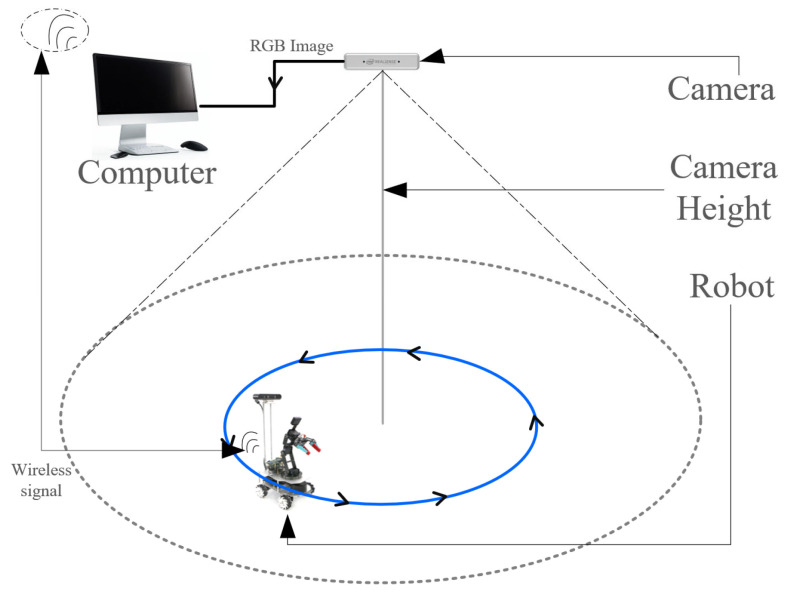
Scenario Diagram of 6D DoF Estimation Experiment Based on the YolPnP-FT Network.

To investigate the impact of camera height on visual localization accuracy—a critical factor in practical deployments—we conduct comparative experiments using a four-axis Mecanum-wheeled robot at three camera heights: 0.5 m, 1.5 m, and 2.5 m. In each setting, the robot executes a standard circular trajectory. The actual and estimated trajectories are compared (Figure 10), with the camera positioned directly above the world coordinate origin and aligned parallel to the ground plane to eliminate coordinate misalignment.

**Figure 11:**
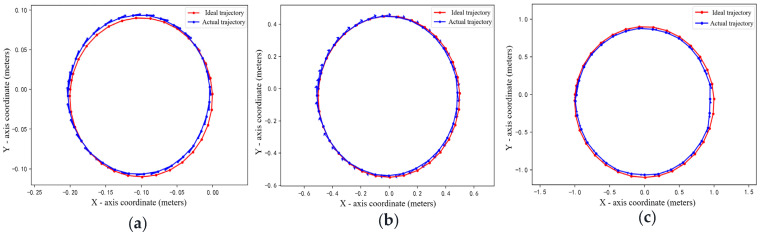
The Difference Between the Actual Circular Motion Trajectories and the Detected Trajectories Under Different Camera Height Settings: (**a**) 0.5 m, (**b**) 1.5 m, and (**c**) 2.5 m.

**Figure 12:**
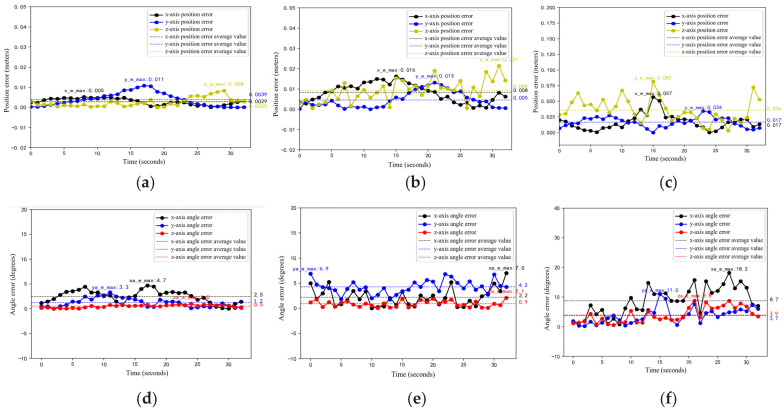
Position (meters) and Angle (degrees) Error Curves for the Robot’s Motion Under Different Camera Heights. (**a**) Positional errors at a height of 0.5 m. (**b**) Positional errors at a height of 1.5 m. (**c**) Positional errors at a height of 2.5 m. (**d**) Angle errors at a height of 0.5 m. (**e**) Angle errors at a height of 1.5 m. (**f**) Angle errors at a height of 2.5 m.

As shown in Figure 11, the detected trajectories closely match the ground-truth circular paths across all heights, with only minor deviations. This indicates that the model provides reliable pose estimates from varying viewpoints. To quantify performance, we compute positional and angular errors between ground-truth and estimated poses (Figure 12).

Table 2 and Table 3 summarize the position and orientation errors along the x, y, and z axes at different camera heights. A clear inverse relationship between camera height and localization accuracy is observed: as height increases from 0.5 m to 2.5 m, both positional and angular errors grow significantly. Specifically, the average *z*-axis position error rises from 0.0025 m to 0.043 m, the average pitch (*y*-axis) angle error increases from 1.2° to 6.5°, and the maximum roll (*x*-axis) angle error surges from 4.7° to 18.2°—consistent with the well-established principle that visual pose estimation accuracy degrades with increasing observation distance.

We attribute this error growth to two primary mechanisms:

(1) Small-object effect: As camera height increases, the robot occupies fewer pixels in the image, causing minor 2D keypoint detection errors to be significantly amplified through PnP triangulation;
(2) Feature degradation: At high (near-top-down) viewpoints, the robot’s upper surface often lacks texture or exhibits symmetry (e.g., planar surfaces), leading to ambiguous keypoint localization. In contrast, lower viewpoints, though more prone to occlusion, provide richer lateral geometric features that enhance matching reliability.

Notably, angular errors exhibit strong directional dependency: yaw (rotation about the *z*-axis) remains relatively stable across heights, as robot orientation can still be inferred from the planar layout of keypoints in the top-down view. In contrast, pitch and roll—dependent on side-view structures—suffer severe degradation at high viewpoints due to feature scarcity.

Considering that a 0.5 m height limits the field of view and hinders multi-robot coverage, we recommend a camera height of 1.5 m, which achieves an optimal trade-off between localization accuracy (average position error: 0.008 m; average angular error: 4.2°) and field-of-view coverage. This height is adopted in all subsequent multi-object tracking experiments.

#### 4.1.3. Comprehensive Real-Time Multi-Robot Formation Tracking Experiments

To further evaluate real-time tracking performance, we conduct a multi-robot formation experiment using one four-axis Mecanum-wheeled robot as the leader and two standard Mecanum-wheeled robots as followers. The leader follows a pre-programmed trajectory, while followers track it using a standard formation control algorithm. The D435i camera captures the formation in real time, and the YolPnP-FT pipeline performs multi-robot recognition, 6D pose estimation, and tracking.

**Figure 13:**
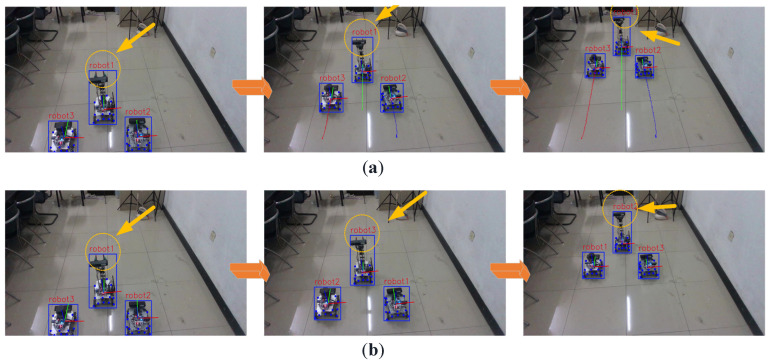
Comparison of Effects with and without the Multi-Target Algorithm. (**a**) The Effect of the Visual Positioning System after Incorporating the Multi-Target Real-Time Tracking Algorithm. (**b**) The Effect of the Visual Positioning System without the Multi-Target Real-Time Tracking Algorithm.

First, we compare tracking performance with and without the multi-object tracking module (Figure 13). Without tracking, the detector assigns IDs randomly in each frame, resulting in severe ID instability. This is attributed to the visual homogeneity among robots, which violates the continuity requirement for reliable multi-robot localization. In contrast, the proposed system, which integrates the adapted DeepSORT tracker, enables consistent ID assignment and significantly improves tracking robustness.

**Figure 14:**
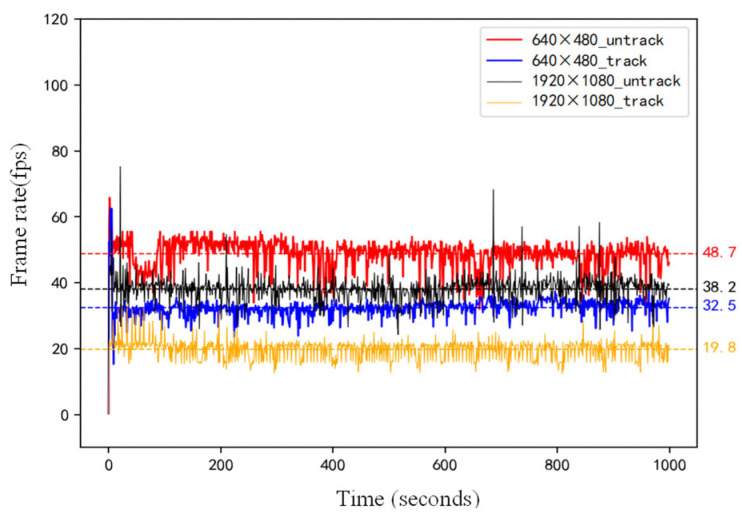
Frame Rate Plots of Multi-Target Real-Time Tracking Based on the YolPnP-FT Under Different Conditions.

Next, we evaluate the impact of input resolution on system throughput. As shown in Figure 14, at 640 × 480 resolution, the system achieves over 30 FPS, satisfying real-time requirements. At 1920 × 1080 resolution, the frame rate reaches 38.2 FPS without tracking and drops to 19.8 FPS with tracking—still sufficient for real-time multi-robot applications.

**Figure 15:**
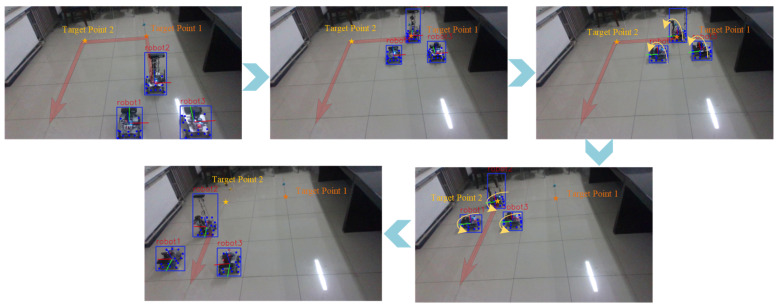
Comprehensive Experimental Process Diagram for Multi-Robot Formation Real-Time Tracking and Positioning.

**Figure 16:**
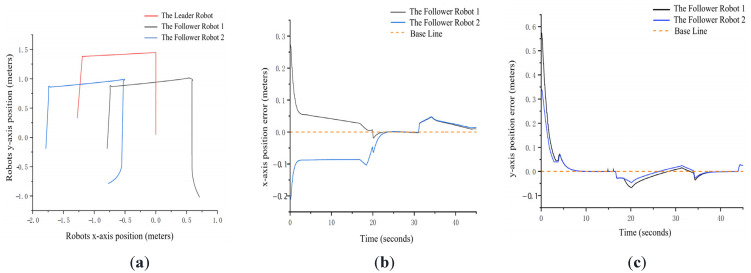
Comparison of Actual and Positioning Trajectories for Multi-Robot Formation Movement. (**a**) Multi-Robot Formation Trajectories; (**b**) Baseline-Relative X-Position Error; (**c**) Baseline-Relative Y-Position Error.

Finally, we perform a comprehensive tracking experiment with heterogeneous robots (Figure 15). Figure 16 compares the actual and estimated trajectories of a follower robot in the x–y plane. Results show that initial errors (up to 0.07 m) gradually converge to below 0.05 m. Notable error spikes at 18 s and 34 s (Figure 16b) are attributed to keypoint occlusion during rotational maneuvers and inertial effects from abrupt stops, respectively. These errors subsequently decay, demonstrating the system’s self-correcting capability.

In conclusion, high-precision 6D pose estimation is necessary but insufficient; stable ID tracking is the prerequisite for multi-robot coordination. Our experiments show that 640 × 480 resolution offers the best balance between computational load and perception quality. For high-precision tasks (e.g., fine manipulation), 1080p resolution can be used at the cost of frame rate. At 19.8 FPS, the system exhibits strong error convergence and robustness to transient disturbances, confirming its readiness for real-world deployment.

### 4.2. Perception-Driven Simulation Validation for Multi-Robot Systems

Section 4.1 validates the accuracy and robustness of the proposed perception system in real-world scenarios. To further demonstrate its practical utility, we inject the real-time 6D pose and identity (ID) outputs from YolPnP-FT into a CoppeliaSim-based simulation environment to drive downstream coordination tasks, including formation control and path planning. It is emphasized that the leader–follower control law and the RRT path planner employed in this section are standard methods and do not constitute contributions of this work. They serve solely as consumers of the perception outputs, thereby verifying whether YolPnP-FT provides sufficiently accurate and stable state estimates to support closed-loop multi-robot coordination.

#### 4.2.1. Multi-Robot Formation Validation

This experiment evaluates whether the ID stability and 6D pose accuracy delivered by YolPnP-FT are sufficient to support formation control in simulation. We deploy five heterogeneous robots in the CoppeliaSim environment and apply a standard leader–follower algorithm. Followers dynamically adjust their relative errors with respect to the leader to maintain formation.

**Figure 17:**
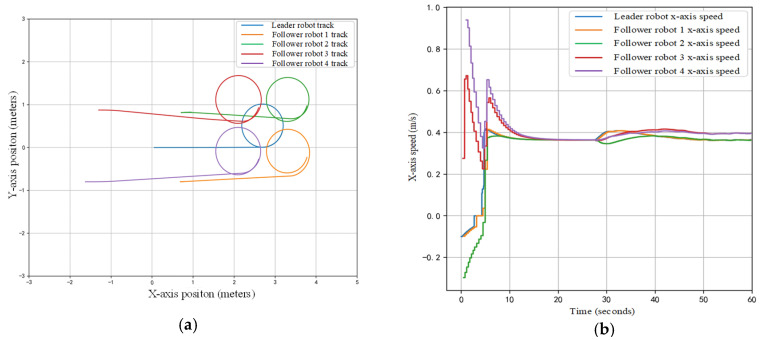
(**a**) Trajectory Plots for Multi-Robots Based on Leader–Follower Algorithm. (**b**) X-Axis Velocity Plot for Multi-Robots Based on Leader–Follower Algorithm.

As shown in Figure 17a, the formation is successfully maintained: followers adjust their velocities to preserve inter-robot spacing. The velocity profile in Figure 17b exhibits a change at 30 s, which corresponds to the leader executing a new trajectory command (e.g., a turn or direction change)—not a perception anomaly. These results confirm that the stable IDs and precise 6D poses provided by YolPnP-FT effectively support the development and validation of formation control algorithms.

#### 4.2.2. Path Planning Validation for Multi-Robot Formations

This experiment assesses whether the 6D pose accuracy from YolPnP-FT is adequate for path planning in simulation. We employ a standard RRT algorithm to evaluate navigation performance.

**Figure 18:**
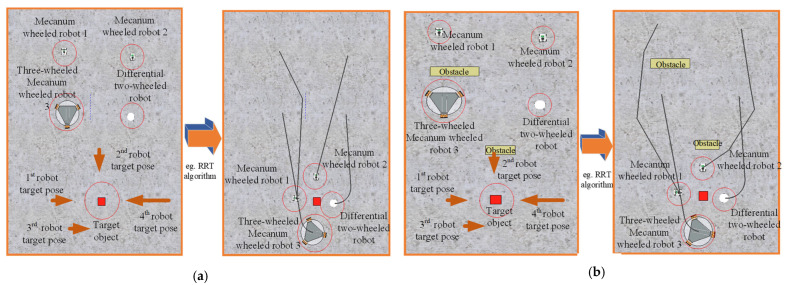
Path Planning for Multi Heterogeneous Robots Based on RRT Algorithm. (**a**) Obstacle-Free Environment; (**b**) Environment with Obstacles.

Figure 18a demonstrates successful navigation in an obstacle-free environment. Figure 18b further validates the system’s capability to avoid static obstacles. The results indicate that the 6D pose information from YolPnP-FT effectively enables path planning for multi-robot formations. Notably, dynamic obstacles are not considered in the current simulation—a limitation to be addressed in future work.

#### 4.2.3. Collaborative Task Validation for Multi-Robot Formations

**Figure 19:**
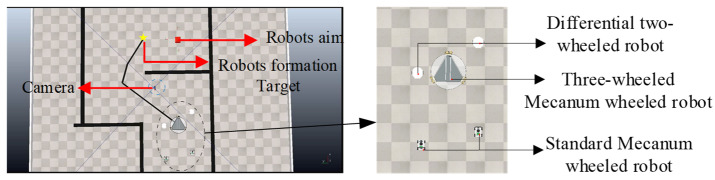
Schematic Diagram for Setting Up a Simulation Scenario of Multi-Robot Formation.

This experiment verifies the utility of YolPnP-FT outputs in complex collaborative tasks within simulation. As illustrated in Figure 19, we construct a simple room environment with an overhead RGB camera. The formation consists of one omnidirectional three-wheeled Mecanum robot (leader), two differential-drive robots, and two standard Mecanum-wheeled robots (followers). The objective is to navigate the formation to a target endpoint using RRT, after which followers autonomously plan paths around the target and orient toward it.

**Figure 20:**
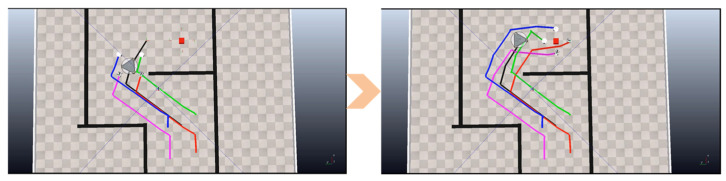
Simulation Verification Process for Multi-Robot Formation Comprehensive Experiment.

During initial simulation runs, the formation collided with room boundaries. Analysis revealed that the RRT planner considered only individual robot poses and ignored the spatial occupancy of the entire formation. Crucially, YolPnP-FT’s complete state output (6D pose + ID for all robots) enabled this diagnostic insight. By redefining the planning reference point as the center of the minimum enclosing circle of the formation and incorporating formation footprint constraints, collisions were eliminated, and the task was successfully completed (Figure 20).

This demonstrates that YolPnP-FT not only provides per-robot states but also supports formation-level algorithm debugging and optimization, significantly reducing trial-and-error costs in real-world deployment.

##### Limitations and Future Work

The current validation assumes static obstacles and a rigid formation model, without considering robot dynamics (e.g., acceleration limits) or interactions with moving obstacles. Additionally, scalability to larger robot fleets (e.g., >10 agents) has not been evaluated. Future work will (1) integrate dynamic obstacle detection, (2) develop real-time replanning frameworks driven by YolPnP-FT outputs, and (3) systematically analyze system throughput and tracking stability as the number of robots increases.”

## 5. Conclusions

This study presents a low-cost, real-time 6D pose estimation and tracking method based on YolPnP-FT perception pipeline for motion capture in multi-robot systems. Using only an Intel RealSense D435i depth camera, several Mecanum-wheeled robots, and a standard host computer, the system achieves simultaneous robot classification, 6D pose estimation, and multi-object tracking in real-world environments. By introducing a keypoint confidence filtering strategy (PnP-FT) and an adapted DeepSORT tracking with cascaded matching mechanism that combines geometric and appearance cues, it effectively mitigates ID switches caused by visual similarity and occlusion. Experimental results demonstrate that, at the recommended camera height of 1.5 m, the system achieves sub-centimeter positional accuracy (average error < 0.009 m) and angular precision (<4.2°), while maintaining robust identity consistency at 19.8 fps with 1080p resolution. Furthermore, the perception outputs are validated in a CoppeliaSim-based simulation environment, confirming their utility for downstream coordination tasks. In summary, this work proposes a low-cost, real-time perception system that provides a practical foundation for affordable and deployable multi-robot algorithms, particularly in visually homogeneous and resource-constrained scenarios.

## Figures and Tables

**Table 1:** Comparison of the advantages of Different Visual Positioning Methods.

Method	6D Pose Est.	6D Pose Track.	Multi Target	Real Time	Hardware Cost	Camera Setup	Env. Constraints
Vicon [1]	Yes	Yes	Yes	Yes	Very High	Multi-camera, fixed	Controlled (no occlusion, stable light)
LOAM [5]	No	No	Limited	Yes	High	LiDAR only	Outdoor/indoor, but needs geometry
GDR-Net [9]	Yes	No	No	No	Medium	Single RGB	Controlled lab
PVN3D [10]	Yes	No	No	Yes	Medium	Single RGB-D	Moderate occlusion OK
FairMOT [11]	No	No	Yes	Yes	Medium	Single RGB	General
DeepSORT [12]	No	No	Yes	Yes	Low	Single RGB	General
Ours	Yes	Yes	Yes	Yes	Low	Single RGB	Indoor, partial occlusion OK

**Table 2:** Position Error Between the Robot’s Actual and Detected Positions Under Different Camera Heights.

Camera Height (m)	Mean X Position Error (m)	Maximum X Position Error (m)	Mean Y Position Error (m)	Maximum Y Position Error (m)	Mean Z Position Error (m)	Maximum Z Position Error (m)
0.5	0.0039	0.005	0.0025	0.011	0.0025	0.008
1.5	0.008	0.016	0.005	0.013	0.009	0.021
2.5	0.017	0.057	0.017	0.034	0.043	0.082

**Table 3:** Angle Error Between the Robot’s Actual and Detected Positions Under Different Camera Heights.

Camera Height (m)	Mean X Angle Error (°)	Maximum X Angle Error (°)	Mean Y Angle Error (°)	Maximum Y Angle Error (°)	Mean Z Angle Error (°)	Maximum Z Angle Error (°)
0.5	2.5	4.7	1.2	3.3	0.5	1.0
1.5	2.2	7.0	4.2	6.9	0.9	2.1
2.5	6.6	18.2	6.5	12.6	3.9	8.8

## Data Availability

The data presented in this study are available on request from the corresponding author due to privacy or ethical restrictions.
